# P385: Microbial contamination of dental unit waterline system (DUWS)

**DOI:** 10.1186/2047-2994-2-S1-P385

**Published:** 2013-06-20

**Authors:** F Abdul Razak, RB Ahmad, WHA Wan Harun, CS Chua

**Affiliations:** 1 Oral Biology, University of Malaya, Kuala Lumpur, Malaysia; 2 University Malaya Dental Center (PPgUM), University of Malaya, Kuala Lumpur, Malaysia

## Introduction

The high presence of microbes in water delivered from dental chair unit (DCU) is of concern as it has been associated with high concentration of endotoxin. The American Dental Association (ADA) recommended a microbial population of 200 cfu/mL as acceptable in DCU water.

## Objectives

The study aimed to evaluate the sanitary level of water from DCUs in a teaching dental clinic and relate the finding to the sterilizing/infection control practices by the clinical personnel.

## Methods

The total microbial load of water samples collected from thirteen DCUs was determined using conventional microbiological methods. Based on the count of colonies formed (cfu) on heterotrophic media plates following an incubation period, the microbial load of the samples was determined in colony forming units per milliliter of water (cfu/mL). Plate counts to determine the presence of pathogenic contaminants such as, total coliforms count, faecal coliforms count, *Escherichia coli* count, faecal streptococci count and *Pseudomonas aeruginosa* were carried out using techniques proposed in the Standard Methods for Examination of Water and Wastewater. The presence of other microorganisms was determined using PCR technique.

## Results

The pH of DCU water in the clinic was found to be slightly acidic at pH 5.4-5.5 and the average water temperature was at 23 °C. The water delivered from all the DCUs was found free of all the pathogens mentioned. The water was however found loaded with other bacteria at varying population: *Sphingomonas rhizogenes* (17.9%), *Sphingomonas dokdonesis* (79.5%), *Sphingomonas mucosissima* (1.1%) and *Methylobacterium radiotolerans* (1.5%). As routine infection control practice, only distilled water is being run in the DCUs. Prior to usage the distilled water was contained in storage bottles before it is dispensed into individual reservoir of each DCU. Since every DCU has bypassed the main connection to municipal water supply, it is thus suggested that the introduction of contaminant may have occurred during storage.

## Conclusion

DCU water in the clinic under study was highly contaminated with microbes and failed to meet recommendation by the ADA. The thorough revise of the infection control practice in the clinic is strongly required.

## Disclosure of interest

None declared.

